# Dynamic single-cell regulomes characterize human peripheral blood innate lymphoid cell subpopulations

**DOI:** 10.1016/j.isci.2023.107728

**Published:** 2023-08-24

**Authors:** Maryline Falquet, Ziyang Su, Tania Wyss, Giuseppe Ercolano, Sara Trabanelli, Camilla Jandus

**Affiliations:** 1 Department of Pathology and Immunology, Faculty of Medicine, University of Geneva, Geneva, Switzerland; 2 Ludwig Institute for Cancer Research, Lausanne Branch, Lausanne, Switzerland; 3 Geneva Center for Inflammation Research, Geneva, Switzerland; 4 Translational Data Science Facility, AGORA Cancer Research Center, SIB Swiss Institute of Bioinformatics, Lausanne, Switzerland; 5 Department of Experimental Pharmacology, University of Naples Federico II, Naples, Italy; 6 Translational Research Center for Oncohematology, Department of Medicine, Faculty of Medicine, University of Geneva, Geneva, Switzerland

**Keywords:** Immunology, Components of the immune system, Cell biology, Transcriptomics

## Abstract

Innate lymphoid cells (ILCs) are plastic immune cells divided into 3 main subsets, characterized by distinct phenotypic and functional profiles. Using single cell approaches, heightened heterogeneity of mouse ILCs has been appreciated, imprinted by tissue signals that shape their transcriptome and epigenome. Intra-subset diversity has also been observed in human ILCs. However, combined transcriptomic and epigenetic analyses of single ILCs in humans are lacking.

Here, we show high transcriptional and epigenetic heterogeneity among human circulating ILCs in healthy individuals. We describe phenotypically distinct subclusters and diverse chromatin accessibility within main ILC populations, compatible with differentially poised states. We validate the use of this healthy donor-based analysis as resource dataset to help inferring ILC changes occurring in disease conditions. Overall, our work provides insights in the complex human ILC biology. We anticipate it to facilitate hypothesis-driven studies in patients, without the need to perform single cell OMICs using precious patients’ material.

## Highlights

• Generation of a single cell RNAseq and ATACseq atlas of human peripheral blood ILCs
• High transcriptional and epigenetic heterogeneity among ILC subsets
• Use of healthy donor-based analyses to infer ILC alternations in disease conditions

## Graphical abstract

**Figure:**
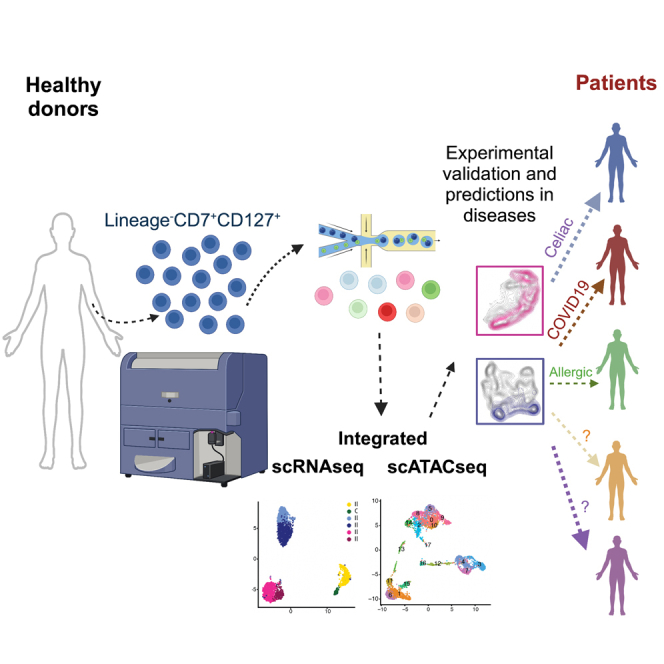

## Introduction

For more than three decades, cytotoxic natural killer (NK) cells have been the only recognized innate lymphocytes. In the past fifteen years, helper innate lymphoid cells (ILCs) have been identified and are now considered as a family of effector cells critical in the regulation of tissue homeostasis and defense.^1^^,^^2^^,^^3^^,^^4^

In human, helper ILCs have been subdivided into 3 main subsets, mirroring the adaptive CD4 T cell helper categorization. ILC1s, ILC2s, and ILC3s were proposed to recapitulate master transcription factor (TF) expression and effector functions of Th1, Th2, and Th17 cells. However, given the absence of an antigen-specific activation of ILCs, their responsiveness to very diverse environmental and microbial-derived stimuli, and their high inter-subset plasticity, this initial classification is emerging as much too simplistic. A number of studies in murine peripheral blood and tissues refined our understanding of ILC heterogeneity within classes,^5^ inter-subset complexity and tissue-specific heterogeneity.^6^ This has been extensively studied in the gut for ILC2s^7^ and ILC3s,^8^^,^^9^ and in the lung for ILC2s.^10^ Tissue-specific signatures and intra-tissue gene program heterogeneity was also shown for murine ILC1s at steady state, but also in disease such as upon tumor development.^11^ Moreover, the combination of single cell (sc)RNA sequencing (seq) and ATACseq analyses on ILCs isolated from different anatomical sites defined core tissue-specific TF mediated regulation of ILC differentiation and function.^8^^,^^9^^,^^12^^,^^13^ While these works shed light on the core transcriptional programs of mouse ILCs, how this regulation occurs in human helper ILCs remains largely unexplored.

In healthy individuals, differentiated ILC1, ILC2, and ILC3 subsets are present in tissues and swiftly react with subset-specific responses upon innate sensing of environmental cues. Beside committed ILC subsets, in the peripheral circulation, a population of ILC progenitors (ILCPs) has also been identified. ILCPs can mature and give rise to the different ILC subsets upon proper cytokine stimulation,^14^ suggesting the existence of a peripheral development to replenish the pool of functionally committed tissue-resident ILC subsets. Further, a strong tissue-specific imprinting has been observed when comparing transcriptomic profiles from thousands of single ILCs across multiple anatomical distributions, such as tonsils, lung, colon, and metabolic tissues.^15^^,^^16^ Similarly, inter-subset conversion appears to be controlled by discrete transcriptomic and epigenetic regulation in different organs *in vivo*, as shown in RNA and chromatin studies of ILC3s transitioning to ILC1s, both in humanized mice and human intestine.^17^ However, whether more complex transcriptional networks and dynamic gene expression changes are responsible for single-cell specific ILC subset heterogeneity, differentiation and plasticity in human remains unexplored.

In this study, we aimed at unraveling the regulatory factors and intra- and inter-subset dynamics of human peripheral ILCs by combining scRNAseq and scATACseq, and *in vitro* validation studies. By providing case-study examples, we propose a scRNAseq and scATACseq resource of ILCs from healthy donors that can be exploited to facilitate the understanding and help in predicting ILC fate decision and function in disease.

## Results

### ILC2s and ILCPs are heterogeneous subsets in the human blood

**Figure 1:**
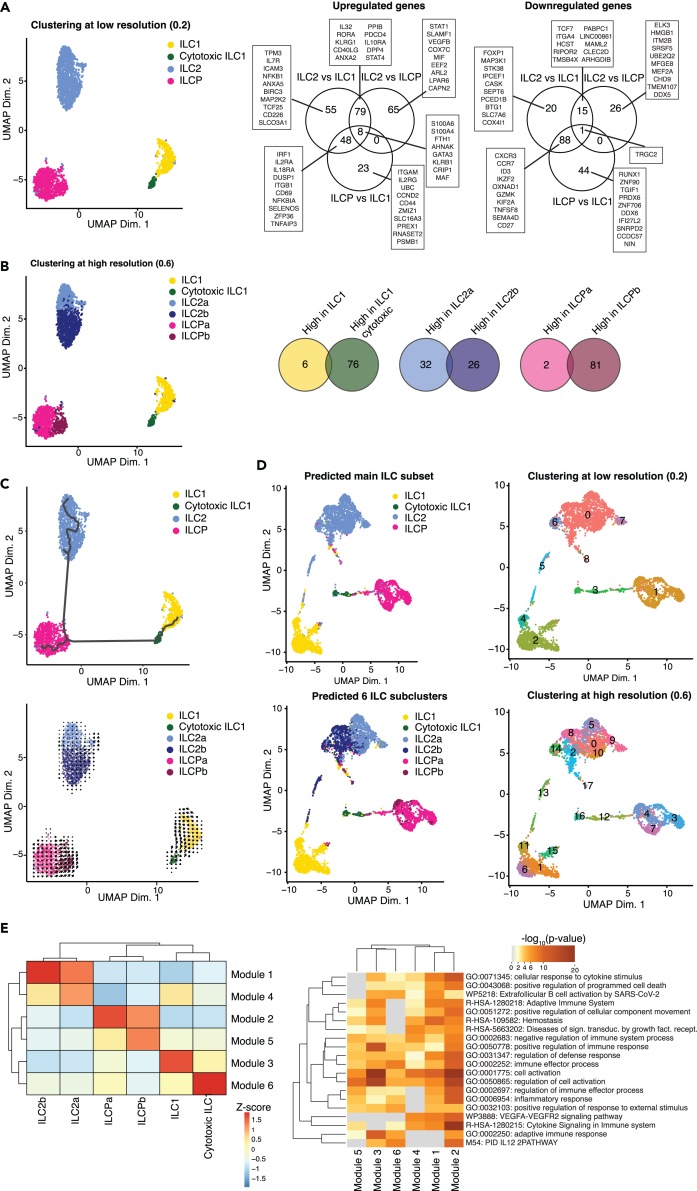
ILC subsets display distinct transcriptional profiles (A) UMAP plot showing clusters of cells annotated to the main ILC subsets from 3 healthy donors. The Venn diagrams show the number of genes up-regulated or down-regulated in all pairwise ILC subset comparisons (excluding mitochondrial or ribosomal genes). Some genes in each comparison are highlighted. See Table S1 for full list of significant genes. (B) UMAP plot showing transcriptionally distinct subpopulations within the main ILC subsets from 3 healthy donors. The Venn diagrams show the number of genes up-regulated or down-regulated in each subpopulation comparison within the ILC subsets (excluding mitochondrial or ribosomal genes). (C) Trajectory (Monocle3) and RNA velocity analyses among ILC subsets, with cells either colored according to ILC subset or subpopulation. (D) UMAP plot of ILCs generated using chromatin accessibility data. Cells are colored either according to label transfer annotation from main ILC subset or subpopulation, or according to cluster identity based on scATACseq data. (E) Heatmap of row Z-scores of gene modules showing different transcriptional profiles across ILC subpopulations (see Table S2 for full list of genes within modules), and heatmap of -log_10_(p value) of over-representation analysis results for genes within each module.

To dissect the heterogeneity of human circulating ILCs, we sorted total ILCs from peripheral blood of healthy donors and performed scRNAseq using the 10x Genomics instrument. We captured a total of 2466 ILCs isolated from 3 donors, of which we retained 2361 high quality cells. By performing unbiased cell clustering analyses at low resolution (0.2) we observed the expected main circulating ILC populations, with highest abundance for ILC2s (1137 cells), followed by ILCPs (809 cells), ILC1s (346 cells), and cytotoxic ILC1s (69) (Figures 1A and S1A). By performing differential gene expression analyses among the identified ILC subsets, we observed a core ILC signature composed of 8 genes that were differentially expressed among all subsets, including *KLRB1* and *GATA3*. Then, a discrete set of genes was specifically up- or down-regulated in each subset, including previously reported ones, such as the upregulation of *RORA*, *KLRG1*, and *CD40LG* in ILC2s compared to the other ILCs, or *GZMK* and *CXCR3* in ILC1s as compared to ILC2s/ILCPs, or IL18RA in ILC2s and ILCPs as opposed to ILC1s. Additionally, other genes not reported previously appeared differentially expressed among subsets, such as *ICAM3* and *VEGFB* that were over-expressed in ILC2s, or *HMBG1* and *MEF2A* in ILCPs as compared to ILC2s (Table S1). Further, clustering at a higher resolution (0.6) revealed a heightened level of intra-subset heterogeneity among ILC2s and ILCPs, but not ILC1s and cytotoxic ILC1s (Figures S1B and S1C), resulting in the presence of a total of 6 clusters. Both ILC2s and ILCPs were subdivided into 2 transcriptionally distinct subpopulations, referred as ILC2a and ILC2b, and ILCPa and ILCPb, respectively, overexpressing between 2 and 81 genes as compared to the other counterpart (Figure 1B and Table S1). To understand the developmental pathways and maturation state relationships of these ILC subclusters, we performed trajectory and RNA velocity analyses (Figure 1C). For trajectory analysis, the start root was selected among ILCPs with low *KIT* expression.^14^^,^^18^ As shown in Figure 1C, bottom panel, the trajectory started among ILCPs, with the ILCPa subcluster emerging as the less mature, then bifurcated either toward ILC2s or ILC1s, confirming that ILCPs are precursors for the other ILC subsets.^14^ The ILC2a subcluster was more distant as compared to the ILC2b one, suggesting for potential different functional properties of these two subpopulations. Further, while cytotoxic ILC1s have been proposed as “terminally differentiated” ILC1s acquiring cytotoxic functions, our pseudo-chronological analyses showed that cytotoxic ILC1s appear to be located at earlier differentiation stages than helper ILC1s, although formal experimental validation through cell differentiation studies would be necessary to confirm this prediction. To evaluate if these transitions are derived from dynamic gene expression mediated by differential chromatin accessibility, we performed scATACseq on ILC sorted from 3 healthy individuals as for scRNAseq (5437 cells captured, 4608 cells retained after removing cells with low number of accessible genomic regions). We used label transfer based on the scRNAseq gene expression profile to infer both the main ILC subsets and the ILC subclusters captured by scATACseq. We identified 1101 ILC1s, 157 cytotoxic ILC1s, 2221 ILC2s, and 1129 ILCPs (Figure 1D), which harbored differentially accessible chromatin regions (Table S2). The main ILC subsets occupied distinct areas within the UMAP generated based on accessible chromatin regions. Clustering of cells based on chromatin accessibility, both at low (0.2) and high resolution (0.6), provided an even more complex layer of diversity showing that within the predicted subclusters additional subpopulations were visible. It also confirmed the proximity of cytotoxic ILC1s to ILCPs, since the clusters that contained them (cluster 12 and 16 at res. 0.6) were very close to ILCP clusters (12, 4, 7, 3). Further, the majority of helper ILC1s and ILC2a distributed among 4 subclusters each, ILC2b distributed mostly among 5 and ILCPa among 3 subclusters. Several intra-subset poised states might favor the rapid expansion and activation of specific ILC subclusters in response to different stimuli, including pathological conditions.

Next, we searched for gene modules that showed distinct expression profiles among the main ILC subsets and subclusters (Figures 1E and S2; Tables S3 and S4). This method identified a module of genes that were enriched in ILC1s (module 3), while cytotoxic ILC1s had elevated expression of genes grouped in a different module (module 6). These modules contained genes involved in different biological pathways, such as IL-12-mediated signaling events (M54) and immune effector process pathway (GO:0002252, Figure 1E, right panel). Increased expression of module 4, with concomitant module 1 gene expression distinguished ILC2a from ILC2b. Both ILCP subsets were enriched in module 2, including VEGFA-VEGFR2 signaling pathway (WP3888), cytokine signaling in immune system, and cellular response to cytokine stimulus pathways (R_HSA-1280215 and GO:0071345), with ILCPb also strongly expressing module 5 gene pathways, highlighting increased cell activation (GO:0001775 and GO:0050865) (Figure 1E, right panel).

### Circulating ILC2s include a tissue-protective and a migratory subset

**Figure 2:**
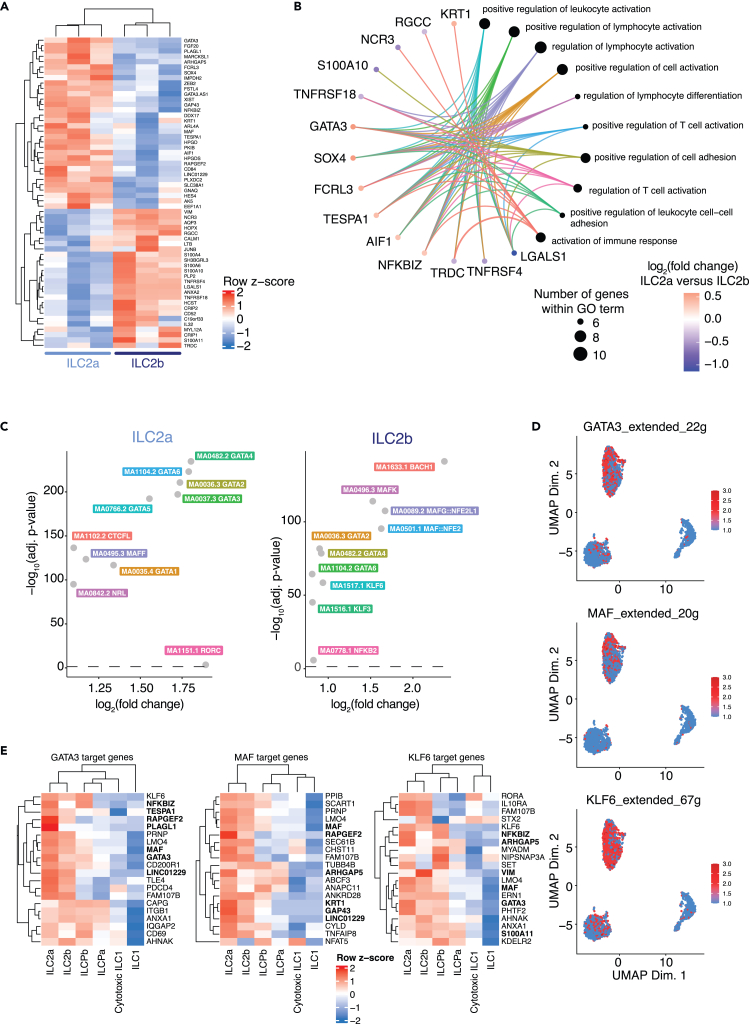
Characterization of two transcriptionally and epigenetically distinct ILC2 subpopulations (A) Heatmap of differentially expressed genes (DEG) between ILC2a and ILC2b subpopulations, averaged per healthy donor (n = 3). (B) Top Gene Ontology gene sets over-represented among DEGs between ILC2a and ILC2b. (C) Transcription factor (TF) motifs differentially accessible in the chromatin of ILC2 subpopulations (chromVar analysis method against the Jaspar TF motif database). (D) Activity of TFs being highly active in ILC2s, inferred from the combination of gene co-expression with *cis*-regulatory motif analysis in scRNAseq (SCENIC). (E) Heatmap of the expression (scRNAseq) of the top target genes of TFs shown in panel D, averaged per ILC subpopulation. The symbols of genes that are significantly differentially expressed between ILC2a and ILC2b are highlighted in bold.

To understand the relationship between ILC2a and ILC2b, we focused on the 32 up- and 26 significantly down-regulated genes between ILC2a and ILC2b (Figure 2A). ILC2a expressed higher levels of *MAF*, *GATA3*, *HPGD*, *HPGDS*, than ILC2b, confirming their more pronounced ILC2 commitment,^19^^,^^20^ as predicted from the trajectory analyses described previously. ILC2b overexpressed *NCR3* (encoding NKp30), *IL32*, *TNFRSF18* (encoding GITR), *LGALS1* (encoding Galectin1), and *LTB*. No difference in terms of memory gene expression was observed when comparing the two subsets, suggesting that ILC2a does not represent an enriched “trained” ILC2 subcluster (Figure S3). To confirm increased protein expression of some of the differentially expressed genes and to identify potential markers to isolate ILC2a and ILC2b, we performed multicolor flow cytometry analyses of ILCs from additional healthy donors. We observed a cluster characterized by higher expression of CD84, GATA-3, and c-Maf, and concomitant lower expression of CD52, NKp30, and OX-40, resembling the ILC2a cluster identified by transcriptomic analysis (Figure S4). Inversely, we identified a cluster with the opposite protein expression profile, like the transcriptomic pattern of ILC2b (Figure S4). When comparing the pathways associated to the up/down-regulated genes in both subsets, we observed that overexpressed genes in ILC2a were generally linked with several Gene Ontology (GO) gene sets involving positive regulation of leukocyte activation, lymphocyte activation, and cell or leukocyte adhesion. Instead, the genes found overexpressed in ILC2b rather converged to one or few pathways associated with immune responses or cell adhesion (e.g., *LGALS1*) (Figures 2B and S2; Table S4). Next, we compared the TF motif enrichment in the open chromatin region of each ILC2 cluster (Figure 2C). We highlighted a pronounced GATA family (i.e., GATA-4, -6, -2, -3, -5) and MAF TF enrichment in ILC2a, while we observed a bias toward BACH1 and KLF family members (i.e., KLF6 and KLF2) in ILC2b. In line with these findings, target genes of these TFs were predominantly expressed in ILC2a (MAF, GATA-3) and in ILC2b (KLF6), respectively, highlighting the activation of the mentioned TFs (Figures 2D and 2E).

### ILCPb display heightened activation compared to quiescent ILCPa

**Figure 3:**
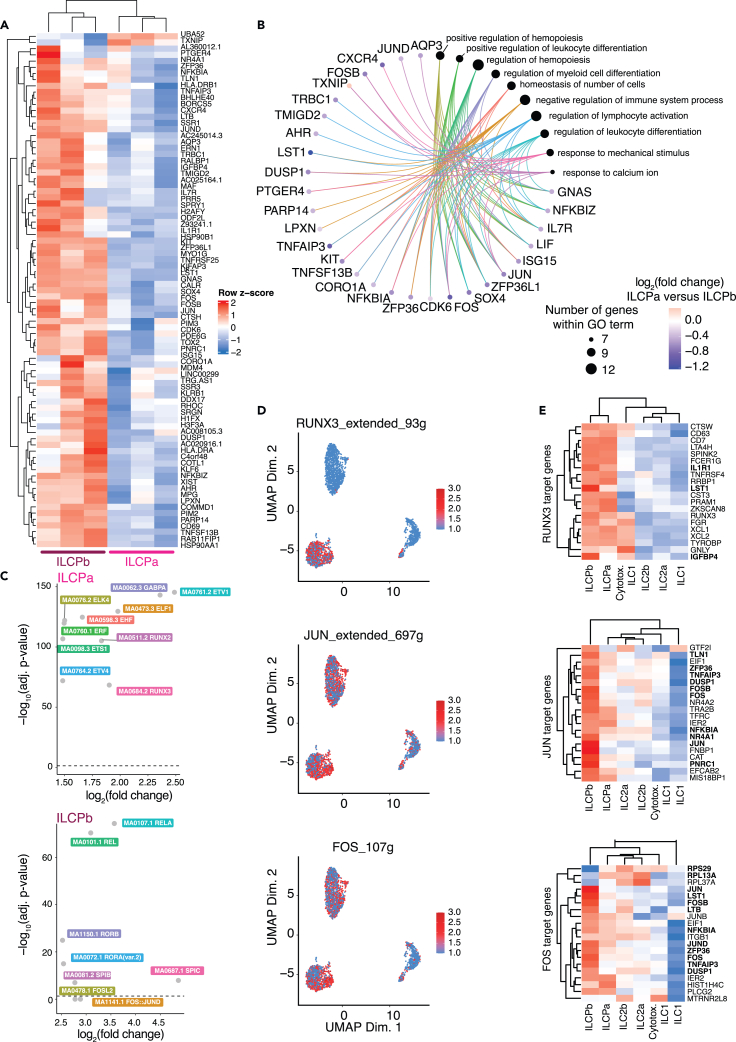
Characterization of two transcriptionally and epigenetically distinct ILCP subpopulations (A) Heatmap of differentially expressed genes (DEG) between ILCPa and ILCPb subpopulations, averaged per healthy donor (n = 3). (B) Top Gene Ontology gene sets over-represented among DEGs between ILCPa and ILCPb. (C) Transcription factor (TF) motifs differentially accessible in the chromatin of ILCP subpopulations (chromVar analysis method against the Jaspar TF motif database). (D) Activity of TFs being highly active in ILCPs, inferred from the combination of gene co-expression with *cis*-regulatory motif analysis in scRNAseq (SCENIC). (E) Heatmap of the expression (scRNAseq) of the top target genes of TFs shown in panel D, averaged per ILC subpopulation. The symbols of genes that are significantly differentially expressed between ILCPa and ILCPb are highlighted in bold.

ILCPa and ILCPb differed from each other by 2 down-regulated and 81 up-regulated genes in the former compared to the latter (Figure 1B). Similarly, the significantly different chromatin regions identified were all less accessible in ILCPa than in ILCPb (Table S2). In line with the trajectory analysis, ILCPa expressed high levels of *UBA52* gene, known to regulate ubiquitination and embryonic development and *TXNIP*, a factor involved in inhibiting thioredoxin activities and in inducing G0/G1 cell-cycle arrest,^21^ confirming their naiver developmental state (Figure 3A). Inversely, ILCPb showed high expression of activation genes such as *CD69*, genes involved in antigen presentation (e.g., *HLA-DRA*, *HLA-DRB*), in chemotaxis (e.g., *CXCR4*, *PGER4*), in interactions with other immune cells, such as B cells (e.g., *TNFSF13B* encoding for BAFF) and in xenobiotic metabolization (e.g., *AHR*). No difference in CD62L transcripts were observed between ILCPa and ILCPb, suggesting that these 2 clusters are distinct from the ILCP subpopulations reported by Kokkinou et al.^22^ To confirm increased protein expression of some of the differentially expressed genes and to identify potential markers to isolate ILCPa and ILCPb, we performed multicolor flow cytometry analyses of ILCs from additional healthy donors. We observed a cluster characterized by lower expression of CD127, cKIT, CD69, CD161, and HLA-DR resembling the ILCPa cluster identified by transcriptomic analysis (Figure S5). Inversely, we identified a cluster with the opposite protein expression profile like the gene profile of ILCPb (Figure S5). Further, in line with the more activated status of ILCPb, GO pathways over-represented in this subpopulation included those of lymphocyte and hemopoiesis regulation and activation, and regulation of leukocyte differentiation (Figure 3B). The comparison of TF motif enrichment in both ILCP subsets showed enrichment for ETS family members in ILCPa (e.g., ETV1, ETS1, RUNX3) and of REL TFs (i.e., REL, RELA) or AP-1 family members (e.g., JUN, FOS, FOSL2) in ILCPb (Figure 3C). Accordingly, the TF motif enrichment in the open chromatin region of each ILCP cluster was increased for these TFs and downstream genes were overexpressed in the expected subset (Figures 3D, 3E, and S2; Table S4).

### Infer disease-relevant regulomes using healthy donor single cell OMICs

Next, we speculated that our single cell multi-omics resource in healthy donors would facilitate prediction of disease relevant ILC-mediated immune alterations. We used three case-studies of previously published data in patients’ cohorts and evaluated our “OMICs” dataset for its ability to infer ILC patients’ changes.

**Figure 4:**
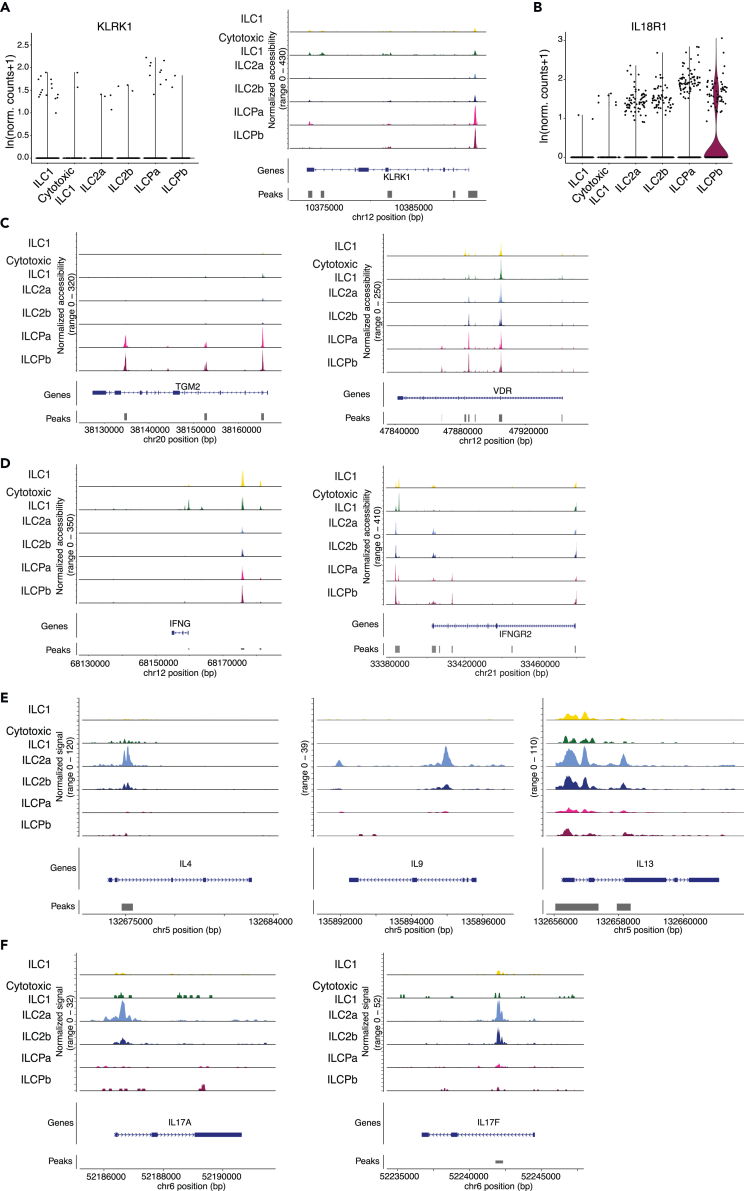
Expression and chromatin accessibility at disease-relevant loci in ILCs (A) Expression (scRNAseq) and chromatin accessibility (scATACseq) of KLRK1 among ILC subpopulations. (B) Expression of IL18R1 among ILC subpopulations (scRNAseq). (C) Genomic coverage at the TGM2 and VDR loci of scATACseq sequencing reads. (D) Genomic coverage at the IFNG and IFNGR2 loci of scATACseq sequencing reads. e Genomic coverage at Type 2 cytokine genes of scATACseq sequencing reads. (F) Genomic coverage at Type 3 cytokine genes of scATACseq sequencing reads. In panels A and C–F, tracks are normalized using a per-group scaling factor computed as the number of cells in the group multiplied by the mean sequencing depth for that group of cells.

As a first case-study we exploited our recent work on COVID-19 infected patients, where we reported the emergence of a population of NKG2D^+^ ILC2s, which correlates with reduced hospitalization time.^23^ As expected, NKG2D transcripts (also referred as *KLRK1*) were absent in human ILC2s in healthy individuals (Figure 4A). However, chromatin accessibility of the NKG2D locus was high in pluripotent ILCPs, as compared to ILC1s and ILC2s, suggesting that these cells might be able to upregulate the receptor under proper stimulation (Figure 4A). We previously showed that, *in vitro*, IL-18 induces NKG2D expression in human ILCs.^23^ Interestingly, the only subset of ILCs that expressed the *IL18R* at mRNA level were ILCPb (Figure 4B), a population that also displayed highly accessible binding sites for Rel/RelA (NF-kB subunits) and Fos/JunD (AP-1 subunits), the two most well-known downstream TFs upon IL-18 stimulation (Figure 3C).^24^ Overall, these observations infer that NKG2D^+^ ILC2s are most likely derived *in vivo* from circulating ILCPb in the context of pro-inflammatory stimulation with IL-18.

As second study-case, we utilized the work on pro-inflammatory ILCPs in the context of celiac disease.^25^ Chromatin accessibility at *TGM2* locus (encoding transglutaminase) was significantly heightened in ILCPs (Figures 4C; Table S2), but mRNA transcripts were undetectable in healthy donors (data now shown). Arguing for the potential responsiveness of these cells to gliadin peptides, leading to pathologic IFN-γ secretion in patients, we observed open chromatin for *IFNGR2 loci* (Figure 4D) and accessible regions upstream of the *IFNG loci* in ILCPs,^26^ in addition to the expected accessibility in ILC1s (Figure 4D). Concomitantly, ILCPs also displayed open chromatin for the vitamin D receptor gene (VDR) (Figure 4C), in line with the VitD property to inhibit IFN-γ secretion by patients’ ILCPs.

As a third study-case, we utilized the work on ILC3-like ILC2s in the context of persistent allergic rhinitis, where increased Type 3 cytokines have been identified in patients’ and mouse ILC2s.^27^ Interestingly, ILC2a scATACseq analyses revealed increased open chromatin of Type 2 cytokine genes (Figure 4E), as expected, but also of IL-17A and IL-17F, that were instead closed in the other ILC subclusters, including both ILCPa and ILCPb (Figure 4F). Therefore, it is tempting to speculate that ILC3-like ILC2s might arise from ILC2a. In line with this hypothesis, it has been shown that in cat allergic patients, ILC2s expressed high levels of CD84, a molecule that we see significantly increased in the ILC2a compared to the ILC2b subcluster.^28^

Altogether, the integrated scRNAseq and scATACseq data of healthy individuals’ peripheral blood ILCs represent a valuable tool to screen for epigenetic variations that might precede changes at transcriptional levels underlying pathological alterations of ILCs in disease, with the limitation that our dataset only captures circulating ILC subpopulations.

## Discussion

Here, we combined scRNAseq and scATACseq to monitor differentiation trajectories in human peripheral blood ILCs. We identified different subclusters within main ILC subsets, defined by distinct transcriptional profiles and chromatin accessibility states. Finally, we utilized our resource to infer about relevant ILC modulations in disease and propose to use our dataset as a framework to help predicting and investigating ILC changes in pathologies, without the need of costly and time-consuming analyses of limited patients’ biological material.

By probing thousands of circulating human ILCs, we report heterogeneity within ILC2s and ILCPs. Initial work on human tonsil ILCs showed intra-subset heterogeneity with the presence of 3 transcriptionally distinct ILC3 populations in this organ (i.e., naive, activated, antigen presenting clusters).^29^ We did not observe these clusters in our dataset, probably due to the peripheral blood origin in contrast to the tissue-origin of the cells used in the other study. Indeed, it is now well accepted that ILC3s fully commit once in tissues, while circulating cKIT^+^CRTH2^-^ ILCs are pluripotent cells sustaining peripheral ILC-poiesis,^14^^,^^30^ upon proper stimuli depending on Notch, IL-23, and RORC.^31^ Regarding ILC2s, it has been recently shown that 2 distinct ILC2 subsets exist in the human peripheral blood and can be distinguished by the expression of cKIT,^19^^,^^20^ that is defining an ILC3-like phenotype. We did not observe significant differences in cKIT expression between ILC2a and ILC2b, while we noted heightened chromatin accessibility for Type 2 and Type 3 cytokines in ILC2a, suggesting that these cells might own plastic features toward ILC3s if stimulated with Type 3 activating pathways.^32^ Specifically, ILC2a might be the source of ILC3-like ILC2s in tissues, in the context of particular diseases (Figure 4). In contrast, ILC2b displayed increased expression of genes linked to regulatory functions (e.g., IL-32, ID3, GITR) and a trend for increased amphiregulin. This observation is in line with previous work showing that amphiregulin^+^ ILC2s that promote tissue repair also express higher levels of NKp30 that is significantly up-regulated in ILC2b as compared to ILC2a in our dataset.^33^

Regarding ILCPs, recent data reported on the existence of peripheral reservoirs for the ILC-poiesis in the peripheral blood^14^ or in tissues,^22^ other than the bone marrow. Both the transcriptional and the epigenetic profiles, as well as confirmatory flow cytometry analyses showed that ILCPa and ILCPb identified in our work do not correspond to these previously described subpouplations (e.g., no difference in CD45RA and CD62L between ILCPa and ILCPb). Rather, they might resemble plastic ILCPs that can convert into ILC1-like cells.

The results of the epigenome analyses in each of these individual cells suggest that although transcriptional heterogeneity has been used to identify rare cell subpopulations, probing epigenetic states might be more powerful in this endeavor. In line with these findings, others have combined transcriptomic and epigenetic profiles at single cell level to successfully infer trajectories and differentiation potential in human fetal hematopoietic development,^34^ in myeloid blasts during acute myeloid leukemia in patients,^35^ or in pluripotent stem cell (iPSC) commitment.^36^

With increasing evidence that ILCs play crucial roles in pathologies ranging from autoimmunity to inflammation and cancer, the use of scRNAseq and scATACseq on human ILCs is expected to help teasing out inter- and intra-subset plasticity underlying disease states. Importantly, while single cell OMICs have been applied in immune and stromal cells directly in patients’ samples, we provide evidence that mechanism-driven hypothesis related to diseases can be successfully probed in healthy individual datasets, such as the resource that we provide here. A limitation of the current framework is that OMICs profiling is not performed in the exact same cell, and that the analyses is exclusively on peripheral blood cells. Implementation of this analysis should be achievable thanks to technological advances allowing to simultaneously probe for transcriptional and epigenetic changes in the same individual cell, also collected from tissues.^37^^,^^38^^,^^39^ Further, while our work provides a snapshot of ILC states in a physiological condition, temporal variables, such as circadian changes, cell adaptation to nutrient uptake, will be captured by extending analyses to time dimensions, by applying recently developed real-time technologies, such as the Life-Seq for transcriptomic analyses.^40^ Whether these temporal analyses will be possible for epigenetic marks at single cell level remains a technology challenge. Collectively, the profiling of human ILCs using scOMIC analyses revealed unexpected intra-subset heterogeneity and poised cell states, potentially preceding pathology-associated changes, as previously proposed for ILC2s in allergic asthma.^41^

### Limitations of the study

We performed scRNAseq and scATACseq only on PBMCs from healthy individuals and used our dataset to infer disease-related changes in gene expression. To further validate the relevance of a given gene in the disease setting and as follow-up study, similar experiments could be performed on patient samples.

## STAR★Methods

### Key resources table

**Table:** 

REAGENT or RESOURCE	SOURCE	IDENTIFIER
**Antibodies**		

FITC anti-human CD3	Beckman Coulter	Cat# 6604623
FITC anti-human CD4	Beckman Coulter	Cat# 6603862
FITC anti-human CD8	Immunotools	Cat# 21810083
FITC anti-human CD14	Beckman Coulter	Cat# 6604110
FITC anti-human CD15	Beckman Coulter	Cat# IM1423U; RRID: AB_131015
FITC anti-human CD16	Beckman Coulter	Cat# IM0814U; RRID: AB_10640417
FITC anti-human CD19	Beckman Coulter	Cat# 6603859
FITC anti-human CD20	Biolegend	Cat# 980202; RRID: AB_2616617
FITC anti-human CD33	Biolegend	Cat# 303304; RRID: AB_314344
FITC anti-human CD34	Biolegend	Cat# 343504; RRID: AB_1731852
FITC anti-human CD94	Miltenyi	Cat# 130-123-678; RRID: AB_2857615
FITC anti-human CD203c	Biolegend	Cat# 324614; RRID: AB_11218991
FITC anti-human FcεRIα	Biolegend	Cat# 334608; RRID: AB_1227653
APC anti-human CD45	Biolegend	Cat# 982304; RRID: AB_2650648
PE-Cyanine anti-human CD7	Biolegend	Cat# 343114; RRID: AB_2563941
Brilliant Violet 421 anti-human CD127	Biolegend	Cat# 351310; RRID: AB_10960140
PerCP-Cyanine 5.5 anti-human CD3	Biolegend	Cat# 300328; RRID: AB_1575008
PerCP-Cyanine 5.5 anti-human CD8	Biolegend	Cat# 344710; RRID: AB_2044010
PerCP-Cyanine 5.5 anti-human CD14	Biolegend	Cat# 367110; RRID: AB_2566712
PerCP-Cyanine 5.5 anti-human CD16	Biolegend	Cat# 302028; RRID: AB_893262
PerCP-Cyanine 5.5 anti-human CD19	Biolegend	Cat# 302230; RRID: AB_2073119
PerCP-Cyanine 5.5 anti-human CD56	Biolegend	Cat# 318322; RRID: AB_893389
Ultra Brilliant Violet 395 anti-human CD45	BD Biosciences	Cat# 563791; RRID: AB_2744400
Brilliant Violet 421 anti-human CD294	BD Biosciences	Cat# 562992; RRID: AB_2737937
Brilliant violet 605 anti-human CD117	Biolegend	Cat# 313218 ; RRID: AB_2562025
PE Dazzle anti-human CD127	Biolegend	Cat# 351336; RRID: AB_2563637
PE anti-human NKp30	Biolegend	Cat# 325208; RRID: AB_756112
PE anti-human c-MAF	Invitrogen	Cat# 12-9855-42; RRID: AB_2572747
Brilliant Violet 510 anti-human CXCR4	Biolegend	Cat# 306536; RRID: AB_2810461
Brilliant Violet 786 anti-human TCRγδ	BD Biosciences	Cat# 740995
Alexa 700 anti-human CD52	R&D Systems	Cat# FAB9889N
APC anti-human CD84	Biolegend	Cat# 326010; RRID: AB_2814188
PE-Cyanine 7 anti-human GATA3	BD Biosciences	Cat# 560405; RRID: AB_1645544
PerCP-Cyanine 5.5 anti-human OX40	Biolegend	Cat# 350010; RRID: AB_10719224
PerCP-Cyanine 5.5 anti-human CD69	Biolegend	Cat# 310926; RRID: AB_2074956
PE-Cyanine 7 anti-human CD161	Biolegend	Cat# 339918; RRID: AB_11126745
Alexa 700 anti-human CD45RA	BD Biosciences	Cat# 560673; RRID: AB_1727496
Brilliant Violet 711 anti-human HLA-DR	Biolegend	Cat# 307644; RRID: AB_2562913

**Biological samples**		

Peripheral Blood Mononuclear Cells (PBMCs) from healthy donors	Local blood transfusion center	N/A

**Chemicals, peptides, and recombinant proteins**		

Zombie UV Fixable Viability Kit	Biolegend	Cat# 423108
Lymphoprep	Promega	Cat# 07801
Puregene RBC Lysis Solution	Qiagen	Cat# 158389
eBioscience ™ Foxp3/Transcription Factor Staining Buffer Set	Invitrogen	Cat# 00-5523-00
DPBS without calcium and magnesium chloride	Sigma	Cat# 8537
BSA	Sigma	Cat# 126609
10x Genomics® Reagent kits	10x Genomics	www.10xgenomics.com

**Deposited data**		

Human tonsil Innate lymphoid cells (ILCs) scRNA-Seq	Björklund et al.^29^	GEO: GSE70580
Genes up-regulated in comparison of memory CD8 T cells versus effector CD8 T cells	Rahimi et al.^42^	GEO: GSE10239
Human peripheral ILCs RNA-Seq	Salomé et al.^43^	ENA: PRJEB34980
Human peripheral ILCs scRNA-Seq and scATAC-Seq (sequencing data)	This paper	GEO: GSE225169
Human peripheral ILCs scRNA-Seq and scATAC-Seq (UMAP and cell annotation)	This paper	Single Cell Portal: SCP2121
Mouse ILCs scRNA-Seq and scATAC-Seq	Verma et al.^44^	GEO: GSE172258

**Software and algorithms**		

FlowJo v10.7.1	BD Biosciences	https://www.flowjo.com
R v4.0.3, v4.1.0 and v4.1.1	The R Project	https://www.r-project.org
Prism v10.0.0	GraphPad	www.graphpad.com
10x Genomics Cell Ranger Pipeline v3.1.0	10x Genomics	www.10xgenomics.com
Seurat v4.0.3 and v4.0.4	Hao et al.^45^	https://cran.r-project.org/web/packages/Seurat/index.html
SingleR v1.0.6	Aran et al.^46^	https://bioconductor.org/packages/release/bioc/html/SingleR.html
clusterProfiler v3.18.1	Yu et al.^47^	https://bioconductor.org/packages/release/bioc/html/clusterProfiler.html
enrichplot v1.10.2	Yu G^47^	https://bioconductor.org/packages/release/bioc/html/enrichplot.html
Monocle3 v1.0.0 in R v4.1.0	Cao et al.^48^	https://cole-trapnell-lab.github.io/monocle3/
velocyto v0.17.17	La Manno et al.^49^	http://velocyto.org/velocyto.py/
velocyto.R v0.6 in R v4.1.1		https://github.com/velocyto-team/velocyto.R
GenomicRanges v1.42.0	Lawrence et al.^50^	https://bioconductor.org/packages/release/bioc/html/GenomicRanges.html
Signac v1.4.0	Stuart et al.^51^	https://cran.r-project.org/web/packages/Signac/index.html
chromVAR v1.14.0	Schep et al.^52^	https://www.bioconductor.org/packages/release/bioc/html/chromVAR.html
Cicero v1.16	Pliner et al.^53^	https://www.bioconductor.org/packages/release/bioc/html/cicero.html
ChIPpeakAnno v3.24.2	Zhu et al.^54^	https://bioconductor.org/packages/release/bioc/html/ChIPpeakAnno.html
SCENIC v1.2.4	Aibar et al.^55^	https://www.aertslab.org/#scenic
Metascape	Zhou et al.^56^	https://metascape.org
ComplexHeatmap v2.6.2	Gu et al.^57^	https://bioconductor.org/packages/release/bioc/html/ComplexHeatmap.html

### Resource availability

#### Lead contact

Further information and requests for resources and reagents should be directed to and will be fulfilled by the lead contact, Camilla Jandus (camilla.jandus@unige.ch).

#### Materials availability

This study did not generate new unique reagents.

### Experimental model and study participant details

Venous blood from HDs was collected at the local blood transfusion center, Lausanne, Switzerland, under the approval of the Lausanne University Hospital’s Institute Review Board, upon written informed consent and in accordance with the Declaration of Helsinki.

### Method details

#### Peripheral blood mononuclear cell (PBMC) isolation

PBMCs were freshly isolated by Lymphoprep (Promega) centrifugation (1800 rpm, 20 min, without break, room temperature). Red blood cell lysis was performed using red blood lysis buffer (Qiagen) and platelets were removed by centrifugation (1000 rpm, 10 min without break, room temperature). Cells were counted and immediately used.

#### Cell sorting and flow cytometry

Human ILCs were identified as lineage negative and interleukin-7 receptor α (CD127) positive lymphocytes. Lineage markers, all FITC-conjugated included: anti-human CD3 (UCHT1, Beckman Coulter (BC), anti-human CD4 (SFCI12T4D11, BC), anti-human CD8 (MEM-31, Immunotools), anti-human CD14 (RMO52, BC), anti-human CD15 (80H5, BC), anti-human CD16 (3G8, BC), anti-human CD19 (J3-119, BC), anti-human CD20 (2H7, Biolegend), anti-human CD33 (HIM3-4, Biolegend), anti-human CD34 (561, Biolegend), anti-human CD94 (Miltenyi Biotech), anti-human CD203c (E-NPP3 Biolegend), anti-human FcεRIα (AER-37, Biolegend). Additional markers used included APC anti-human CD45 (HI30, Biolegend), PE-Cyanine 7 anti-human CD7 (CD7-6B7, Biolegend), Brilliant Violet 421 anti-human CD127 (A019D5, Biolegend), PerCP-Cyanine 5.5 anti-human CD3 (UCHT1, Biolegend), PerCP-Cyanine 5.5 anti-human CD8 (SK1, Biolegend), PerCP-Cyanine 5.5 anti-human CD14 (HCD14, Biolegend), PerCP-Cyanine 5.5 anti-human CD16 (3G8, Biolegend) and PerCP-Cyanine 5.5 anti-human CD19 (H1B19, Biolegend), PerCP-Cyanine 5.5 anti-human CD56 (HCD56, Biolegend). Total ILCs were sorted to at least 98% purity using FACSAria (Becton Dickinson) (Gating strategy shown in Figure S6). By adding anti-CD4 and anti-CD8 antibodies in the lineage cocktail we are aware that ILCs expressing CD4/CD8, described in,^58^ were excluded from the analyses.

For experimental validation, additional markers included: Ultra Brilliant Violet 395 anti-human CD45 (HI30, BD Biosciences), Brilliant Violet 421 anti-human CD294 (CRTH2) (BM16, BD Biosciences), Brilliant Violet 605 anti-human CD117 (c-Kit) (104D2, Biolegend), PE Dazzle anti-human CD127 (A019D5, Biolegend), PE anti-human NCR3 (NKp30) (P30-15, Biolegend), PE anti-human c-MAF (symOF1, Invitrogen), Brilliant Violet 510 anti-human CXCR4 (12G5, Biolegend), Brilliant Violet 786 anti-human TCR*γ*δ (11F2, BD Biosciences), Alexa 700 anti-human CD52 (Hu116, R&D), APC anti-human CD84 (Biolegend), PE-Cyanine 7 anti-human GATA3 (L50-823, BD Biosciences), PerCP-Cyanine 5.5 anti-human OX40 (Ber-ACT35, Biolegend), PerCP-Cyanine 5.5 anti-human CD69 (FN50, Biolegend), PE-Cyanine 7 anti-human CD161 (HP-3G10, Biolegend), Alexa 700 anti-human CD45RA (HI100, BD Biosciences) and Brilliant Violet 711 anti-human HLA-DR (L243, Biolegend). Cells were first stained with Zombie UV Fixable Viability Kit to exclude dead cells, followed by a cocktail of surface antibodies for 20 min at room temperature. For intracellular staining, cells were first fixed and permeabilized with eBioscience Foxp3/ Transcription Factor Staining Buffer Set (Invitrogen). Data were acquired on LSRFortessa flow cytometer and analyzed using FlowJo software (v10.7.1).

FlowJo and the plugin FlowSOM installed in R were used for data downscaling, clustering, dimensionality reduction, analyses and visualization. The compensation matrix was generated based on single-marker samples that were acquired for each experiment. After data compensation, cells were gated as Lin^−^CD127^+^CRTH2^+^ (for ILC2s) or Lin^−^CD127^+^CRTH2^−^cKIT^+^ (for ILCPs), scaled down, concatenated. Uniform Manifold Approximation and Projection (UMAP) followed by clustering using FlowSOM (Self-Organizing Map) plugin were performed on all markers and the generated UMAP were visualized using ClusterExplorer plugin.

#### Sample preparation for single cell RNA sequencing

Total peripheral ILCs were sorted as CD45^+^Lin^−^CD127^+^CD7^+^. Sorted cells were washed with PBS 0.04% Bovin Serum Albumin (BSA) and processed using Chromium Single Cell 3′ v3 Reagent kits according to the manufacturer’s protocol from 10x Genomics. scRNAseq libraries were sequenced on an Illumina NovaSeq 6000.

#### Sample preparation for single cell ATAC sequencing

Total peripheral ILCs were sorted as CD45^+^Lin^−^CD127^+^CD7^+^ and sorted cells were washed with PBS 0.04% Bovin Serum Albumin (BSA). Nuclei isolation was performed according to the manufacturer’s protocol from 10x Genomics (protocol named Nuclei Isolation for Single Cell ATAC Sequencing) using the Chromium Next GEM Single Cell ATAC Reagent Kits v1.1.

#### Single cell RNA sequencing data analysis

Cells were called, and reads per gene per cell were summarized using the 10x Genomics Cell Ranger Pipeline (v3.1.0), using default parameters. Filtered feature per cell barcode matrices generated by cellranger were imported into R (v4.0.3) and pre-processed using the Seurat package (v4.0.3).^45^ We removed cells containing too few (<500) or too many (>2000) detected genes. Too few genes represent low-quality cells or empty droplets, while too many may represent droplets with multiple cells. Cells with excessive mitochondrial gene expression level (>20%) were also excluded, as low quality/dead cells often exhibited extensive mitochondrial contamination. Cells with excessive (>6%) dissociation-related gene expression level were also removed. Removal of biases due to donor origin (batch effect removal) was performed using Seurat’s MultiCCA sample alignment. The subset of genes that showed high variation in each individual dataset (i.e., they are strongly expressed in some cells and very low in others) were selected using parameters selection.method = "vst", nfeatures = 2000. These genes were used to identify anchors with the FindIntegrationAnchors() function, followed by integration of all 3 healthy donors together with the IntegrateData function (dims = 30).

#### Dimensionality reduction and clustering

PCA was constructed on the integrated dataset based on the scaled data of the top 2000 highly variable genes selected by the variance stabilizing method. Clustering and UMAP visualization were performed using 15 principal components and resolutions of 0.2 and 0.6 for the shared nearest neighbor clustering algorithm implemented in the FindNeighbors (k.param = 20) and FindClusters() function.

#### ILC subset annotation

We used several methods to identify ILC subsets within the scRNAseq dataset:

(i) We generated violin plots for known ILC subset marker genes and determined in which cluster each gene is expressed the most (e.g., Figure S1A).
(ii) We calculated gene signature scores for lists of genes expressed in ILC1s, ILC2s, ILCPs and NK cells, as defined by Bjorklund et al.^29^ using the AddModuleScore function of the Seurat package for each of our ILCs at the single-cell level. Briefly, the mean expression level of each gene in the defined expression profiles was calculated for each cell, and the aggregated expression for a set of control genes was then subtracted. The genes in each signature were binned based on the mean expression level, and the control genes were randomly selected from each bin. Violin plots were used to assess the distribution of module scores for each ILC gene signature within each cluster of cells.
(iii) The transcriptomics profiles of each single cell was correlated to the transcriptome of known ILC subsets using SingleR (v1.0.6)^46^ and our RNA seq data from ILC1s, ILC2s, ILCPs and cytotoxic ILCs from Salomé et al. 2019^43^ as a reference. First, the Spearman coefficient of single cells is calculated against each sample in the reference dataset. Correlation analysis was performed only on hypervariable genes identified in the reference dataset. Several runs of correlations were performed by SingleR until the label for each cell was assigned according to the top correlation score against the reference ILC subset.

#### Differential expression analysis and GO enrichment analysis

To extract the main cluster markers at resolution 0.2, the Wilcoxon Rank-Sum test implemented in FindAllMarkers was executed with logfc.threshold = 0.15 and min.pct 0.1. For differential gene expression between pairs of subclusters identified at resolution 0.6, FindMarkers was applied with logfc.threshold set to 0.15. Genes were filtered based on Benjamini-Hochberg-adjusted p value <0.05. Over-represented analysis of Biological Process Gene Ontology (GO) terms (obtained from The Molecular Signature Database v7.1) for the differentially expressed genes was performed using the enricher function of the clusterProfiler package for R (v3.18.1),^47^ separately for the up- or down-regulated genes. Gene-Concept network plots of the top GO terms were generated using the cnetplot function of the enrichplot package for R (v1.10.2).

#### Memory signature analysis in ILC2s

To determine whether ILC2a and ILC2b differed in the expression of memory-related genes, we calculated a score of memory signatures using the AddModuleScore function of the Seurat package. The first signature consisted of a combination of genes up-regulated in memory versus naive CD8 T cells obtained from the Molecular Signatures Database (https://www.gsea-msigdb.org/gsea/msigdb/human/geneset/GSE10239_NAIVE_VS_MEMORY_CD8_TCELL_DN.html) and genes up-regulated in ST2^+^ Th2 cells (obtained from Figure 5E from Rahimi et al.^42^). The other signatures consisted of genes up- or down-regulated in memory ILC2s induced in murine lungs in the context of asthma (obtained from Figure 5C from Verma et al.^44^).

#### Trajectory analysis using monocle 3 and RNA velocity analysis

We followed the pipeline described in the online documentation (https://github.com/satijalab/seurat-wrappers/blob/master/docs/monocle3.html) to construct a Monocle3 (v1.0.0 in R v4.1.0)^48^ object that harbored the UMAP we generated as described above using Seurat functions. After graph learning was performed (learn_graph), the cells were ordered using order_cells() by setting a starting node embedded in the ILCP subcluster 4 with high expression level of CD117. All the trajectory graphs were visualized using the plot_cells() function with or without a trajectory graph.

Monocle 3 offers several approaches for differential expression analyses using regression or graph-autocorrelation. In this study, we identified genes co-regulated along the pseudotim by graph-autocorrelation and modularized them. To detect co-regulated genes, the graph-autocorrelation function graph_test() was specified with a “principal_graph” parameter and significant genes were selected (q value <0.001). Modularization was performed using find_gene_modules() with default parameters and a resolution of 0.001. Module genes were characterized by the enrichment analysis with Biological Process GO terms or KEGG pathways using the Multi-list Enrichment Analysis Pipeline of the online tool Metascape (https://metascape.org).^56^ The p values were calculated based on the cumulative hypergeometric distribution. Given three gene lists A, B and C, p values are always calculated on A, B, and C alone, and on A + B + C combined. The lowest p value out of the four was kept for reporting.

RNA velocity analysis was performed by first estimating the number of spliced and unspliced mRNAs per gene using the run10x function of the python implementation of velocyto (v0.17.17).^49^ Loom files were imported into R (v4.1.1) and RNA velocities were calculated using the velocyto.R package (v0.6).

#### Investigation of transcription factor activity

To predict transcription factor (TF) regulatory networks in ILCs from their scRNAseq profile, we performed SCENIC (v1.2.4)^55^ analyses on the integrated (batch corrected) dataset. We followed the general SCENIC workflow from gene filtration to binarization of transcription factor activity, as described by the authors (https://www.aertslab.org/#scenic), using a provided cis-Target reference based on *hg38*. We then added the regulonAUC matrix as an assay in the Seurat object to ease visualization and relate them to our previous analysis. The SCENIC tool provides a database of TF target genes. In the SCENIC workflow, co-expression between TF and target genes is first inferred using GENIE3.^59^ The next step involves the R package RcisTarget, which evaluates TF binding site motif enrichment in the vicinity of transcription start sites of target genes. These steps allow to predict TF-target gene links, which are then summarized into a “regulon” activity score using AUCell.^55^ For each target gene, a Spearman correlation score is calculated. For the TFs we found as differentially active among ILC subsets, we extracted the target genes, selected the 20 target genes with highest Spearman correlation score, calculated their average expression per donor, and produced a heatmap using the ComplexHeatmap (v2.6.2)^57^ for R.

#### Single-cell ATAC sequencing data analysis

Cells were called and chromatin accessibility peaks were detected using the “cellranger-atac count” function of 10x Cellranger pipeline (genome reference: GRch38) for each sample independently. Because a different peak set was detected for each sample, the reduce function of GenomicRanges package (v1.42.0)^50^ was used to combine the peaks into a unified peak set. Peaks with width greater than 5000 or less than 10 nucleotides were removed.

#### Downstream analysis of scATACseq data

The downstream analysis was done in R (v4.1.0) using Seurat (v4.0.4), Signac (v1.4.0),^51^ and chromVAR (v1.14.0).^52^ The pipeline included a QC step: we retained cells with more than 3000 peaks, more than 50% reads in peaks and less than 0.3% reads in blacklisted regions. Peaks were annotated to genes using the GetGRangesFromEnsDb function of the Signac package and the EnsDb.Hsapiens.v86 annotation. The cells originating from the 3 healthy donors were integrated with the FindIntegrationAnchors and IntegrateData function with dim = 2:30. Dimensions were first reduced with partial singular value decomposition after integration (RunSVD function with n = 30), followed by Uniform Manifold Approximation and Projection dimensional reduction (RunUMAP function with dims = 2:30), and finally clustering of cells (FindNeighbors function with dims = 2:30 and resolution = 0.2 and 0.6).

TF activities on the ATACseq data were calculated using the Signac wrapper of chromVAR using the RunChromVAR function, obtaining the TF motifs from JASPAR2020. Significant differences in motif activities among ILC subsets were calculated using the FindMarkers function. Cicero (v1.16)^53^ was used to infer gene-activity scores by linking distally correlated ATAC peaks to gene promoter peaks. For each gene, we computed the Pearson correlation coefficient r between the gene expression and the accessibility of each peak within 500 kb of the gene’s TSS. For each peak, we then computed a background set of expected correlation coefficients given properties of the peak by randomly sampling 200 peaks located on a different chromosome to the gene, matched for GC content, accessibility, and sequence length (MatchRegionStats function in Signac). We then computed the Pearson correlation between the expression of the gene and the set of background peaks. A *Z* score was computed for each peak as z = (r − μ)/σ, where μ was the background mean correlation coefficient and σ was the standard deviation of the background correlation coefficients for the peak. We computed a p value for each peak using a one-sided z-test and retained peak-gene links with a p value < 0.05 and a Pearson correlation coefficient > 0.05 or < −0.05. This was performed using the LinkPeaks function in Signac.^51^

#### Label transfer of ILC subset and subcluster from scRNAseq to scATACseq data

The ILC subsets inferred using the scRNAseq data were predicted in the scATACseq data according to the gene activity matrix generated from scATACseq data. First, the "gene activity" assay of the scATACseq data were scaled and normalized. We used our scRNAseq data as a reference dataset then applied the Seurat function FindTransferAnchors on the Canonical Correlation Analysis (CCA) space for capturing the shared feature correlation structure between scRNAseq and scATACseq data. The cell types were assigned to the scATACseq cells by applying the Seurat TransferData on the 2:30 LSI components, using as cell type label either IDs at resolution 0.2 or at resolution 0.6 of the scRNAseq data. Finally, peaks differentially accessible among ILC clusters or subsets were assessed using the FindAllMarkers and FindMarkers functions of the Seurat package, using parameters min.pct = 1, test.use = LR, latent.vars = peak.region.fragments. Significant peaks were annotated to the closest gene using the annoPeaks function of the ChIPpeakAnno package (v3.24.2),^54^ with parameters bindingType = fullRANge, bindingRegion = c(-10000, 5000), select = bestOne and genome annotation EnsDb.Hsapiens.v86.

### Quantification and statistical analysis

The statistical methods used are related to the bioinformatics analyses of the data, and are fully described in the above sections. The bioinformatics functions and their parameters are listed in the above sections. Significance threshold level was set to p value < 0.05, and p value adjustment was performed to correct for multiple comparisons, as indicated above in the methods sections.

## Data Availability

The sequencing data and raw count matrices have been deposited on Gene Expression Omnibus: GSE225169 and are publicly available as of the date of publication. Normalized data, ILC annotations and UMAPs of the scRNAseq and scATACseq data have been deposited on the Single Cell Portal: SCP2121. Accession numbers are listed in the key resources table. This paper also analyzes existing, publicly available data. The accession number of these datasets are listed in the key resources table. Any additional information required to reanalyze the data reported in this paper is available from the lead contact upon request.

## Supplemental information

Document S1. Figures S1–S6

Table S1. Differentially expressed genes among ILC clusters or among ILC subsets (scRNAseq), related to Figures 1 and 2

Table S2. Differentially accessible chromatin regions among ILC clusters or among ILC subsets (scATACseq), related to Figures 1, 2 and 3

Table S3. List of genes included in gene modules, related to Figure 1

Table S4. Over-representation analysis of Gene Ontology terms using differentially expressed genes between ILC subclusters, related to Figure 3
