# Supplementary material for: Dynamic single-cell regulomes characterize human peripheral blood innate lymphoid cell subpopulations
Source: iScience. 2023 Aug 24;26(9):107728. doi: 10.1016/j.isci.2023.107728 (PMC10483052; doi:10.1016/j.isci.2023.107728)
Supplement: Document S1. Figures S1–S6 [file mmc1.pdf]

iScience, Volume *26*

Supplemental information

Dynamic single-cell regulomes characterize human peripheral blood innate lymphoid cell subpopulations

Maryline Falquet, Ziyang Su, Tania Wyss, Giuseppe Ercolano, Sara Trabanelli, and Camilla Jandus

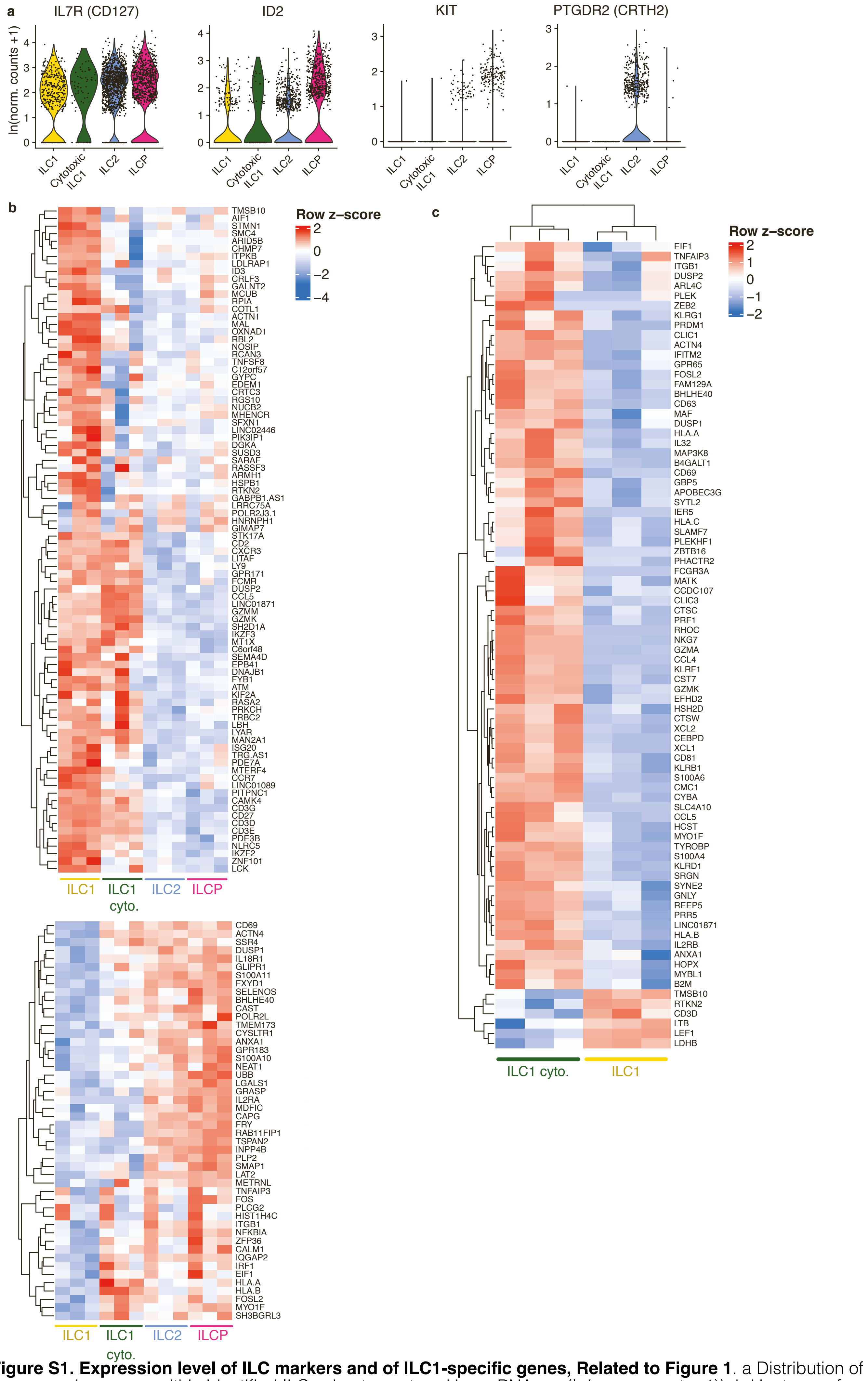

Figure S1. Expression level of ILC markers and of ILC1-specific genes, Related to Figure 1. a Distribution of the expression of known marker genes within identified ILC subsets captured by scRNAseq (ln(norm. counts+1)). b Heatmap of z-scores of genes differentially expressed in ILC1 vs ILC2 and in ILC1 vs ILCP, averaged per healthy donor (n=3, scRNAseq). The top heatmap shows genes up-regulated in ILC1, while the bottom heatmap shows genes down-regulated in ILC1. Related to Figure 1a. c Heatmap of genes differentially expressed between ILC1 and cytotoxic ILC1, averaged per healthy donor (n=3, scRNAseq). Related to Figure 1b.

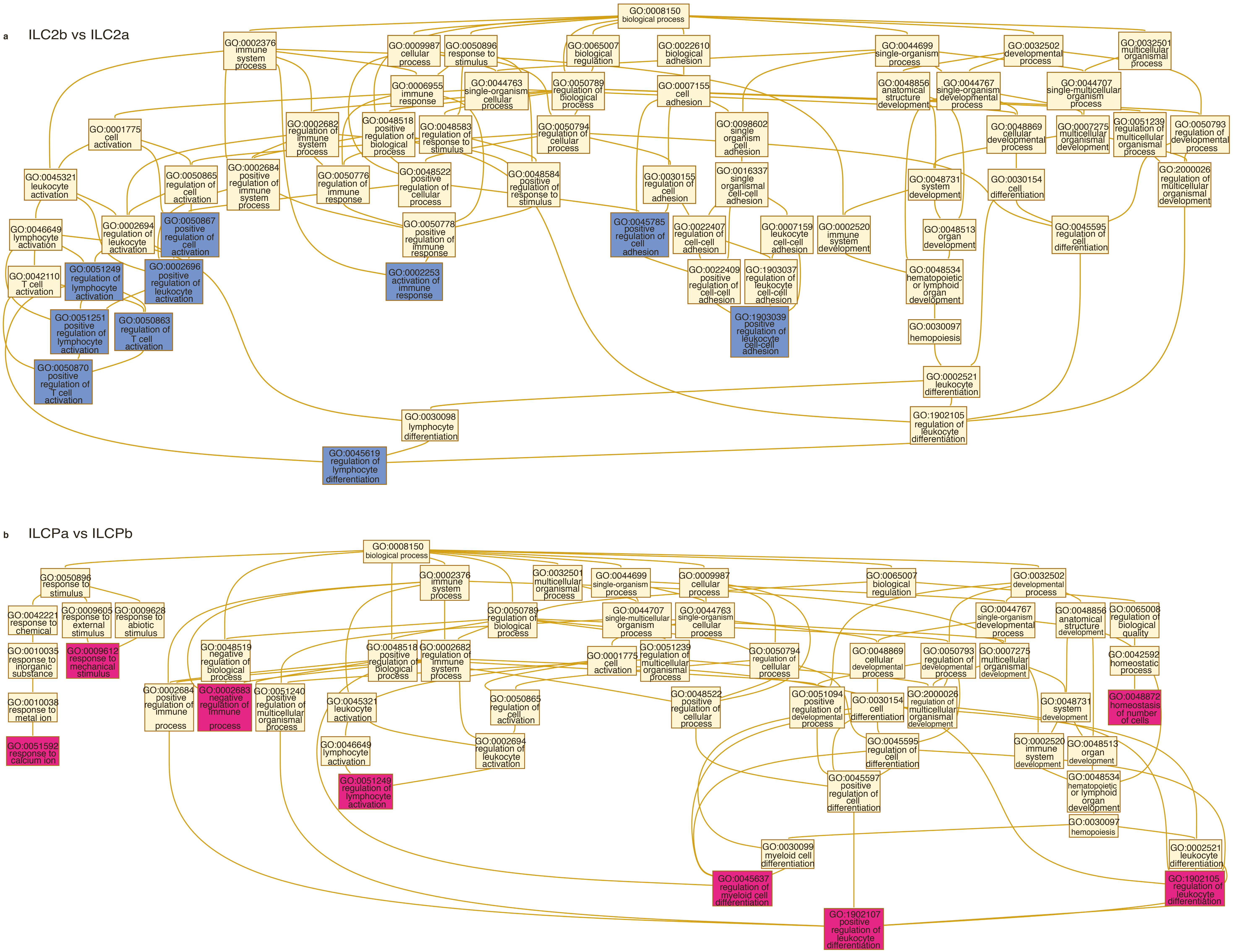

Figure S2. Directed acyclic graphs showing similarities among Gene Ontology (GO) terms, Related to Figures 2 and 3. a GO terms significantly enriched among genes differentially expressed between ILC2a and ILC2b (labelled in blue), showing their relationship with other related GO terms. The full list of significant terms is listed in Supplementary Table 4. b GO terms significantly enriched among genes differentially expressed between ILCPa and ILCPb (labelled in pink), showing their relationship with other related GO terms. The full list of significant terms is listed in Supplementary Table 4. These graphs were produced using the GOView tool of WebGestalt (https://www.webgestalt.org/2017/GOView/).

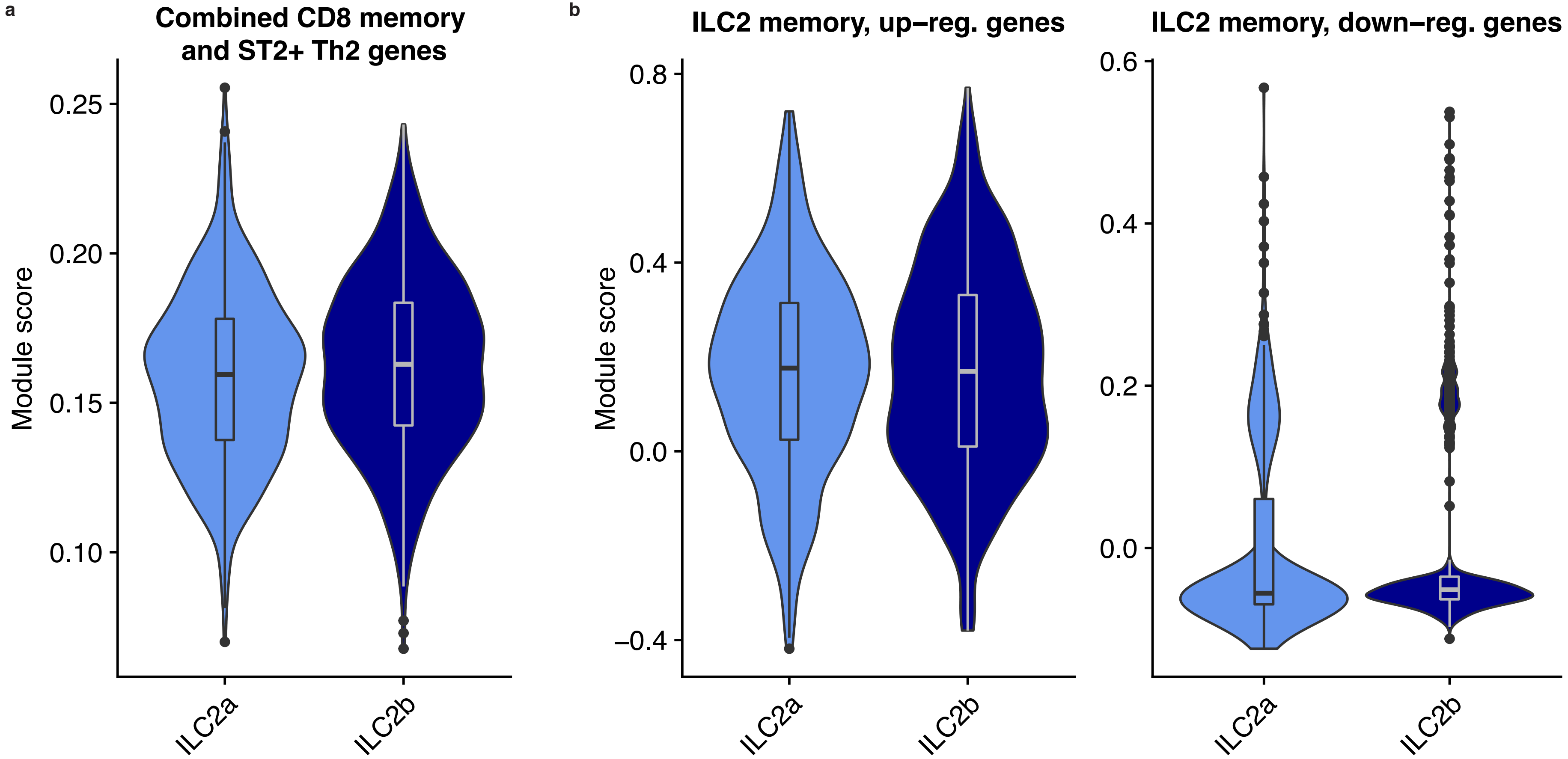

Figure S3. Distribution of memory signature scores within ILC2 subpopulations, Related to Figure 2. a Module score of memory genes extracted from a CD8 T cell memory signature and genes up-regulated in ST2+ Th2 cells (see Methods). b Module score of memory genes up-regulated or down-regulated in mouse ILC2s exposed to asthma (see Methods).

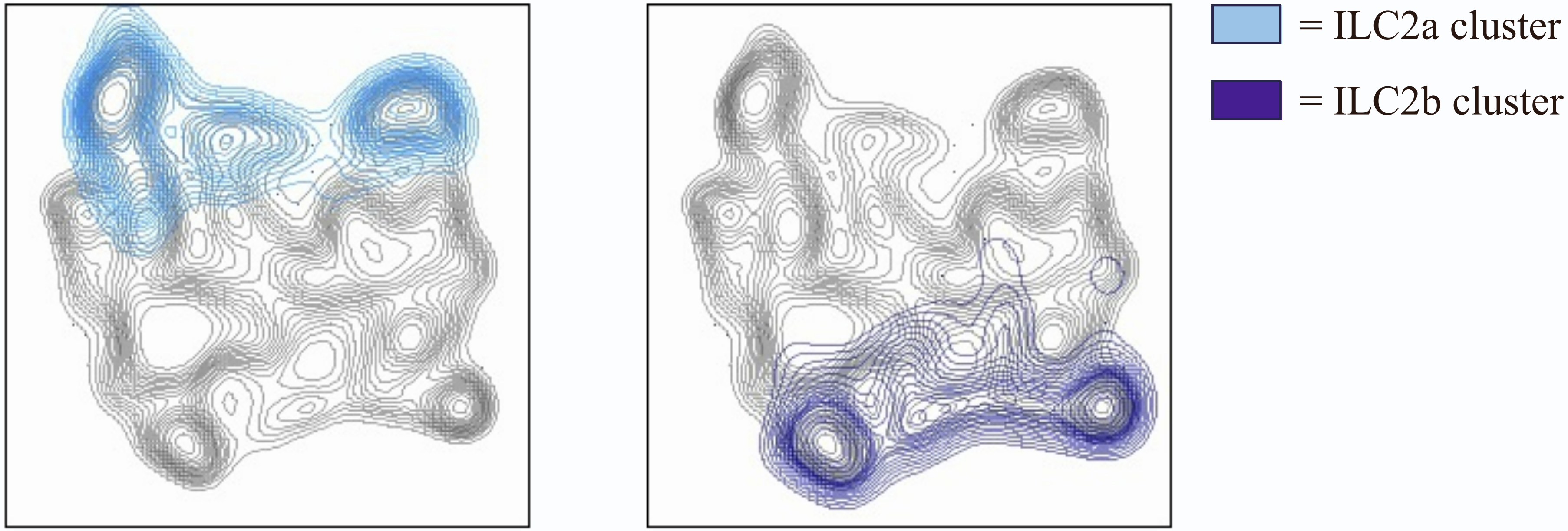

Figure S4. UMAP dimensionality reduction analysis of PBMCs from HD ILC2s, Related to Figure 2. ILC2a cluster was identified based on Lin-CD127+CRTH2+CD84highCD52lowNKp30high phenotype, ILC2b cluster was identified based on Lin-CD127+CRTH2+CD84lowCD52highNKp30low phenotype (n=3 HDs).

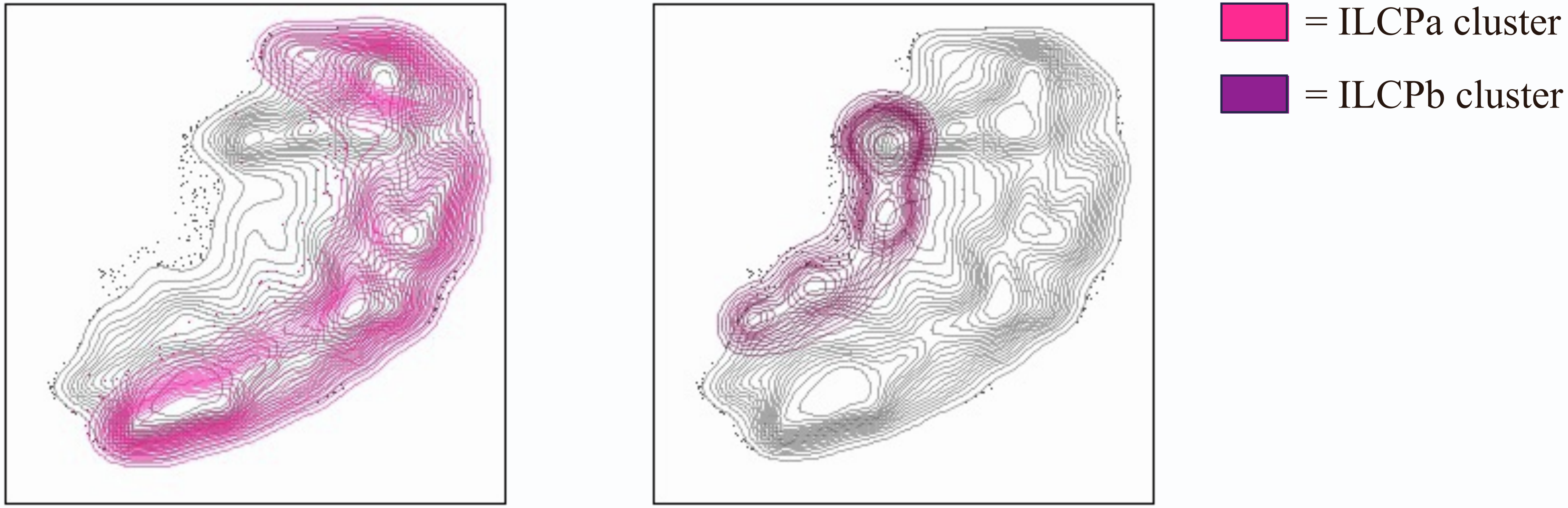

Figure S5. UMAP dimensionality reduction analysis of PBMCs from HD ILCPs, Related to Figure 3. ILCPa cluster was identified based on Lin-CD127lowcKITlowCD69lowCD161low HLA-DRlow phenotype, ILCPb cluster was identified based on Lin-CD127highcKIThighCD69highCD161high HLA-DRhigh phenotype (n=3 HDs).

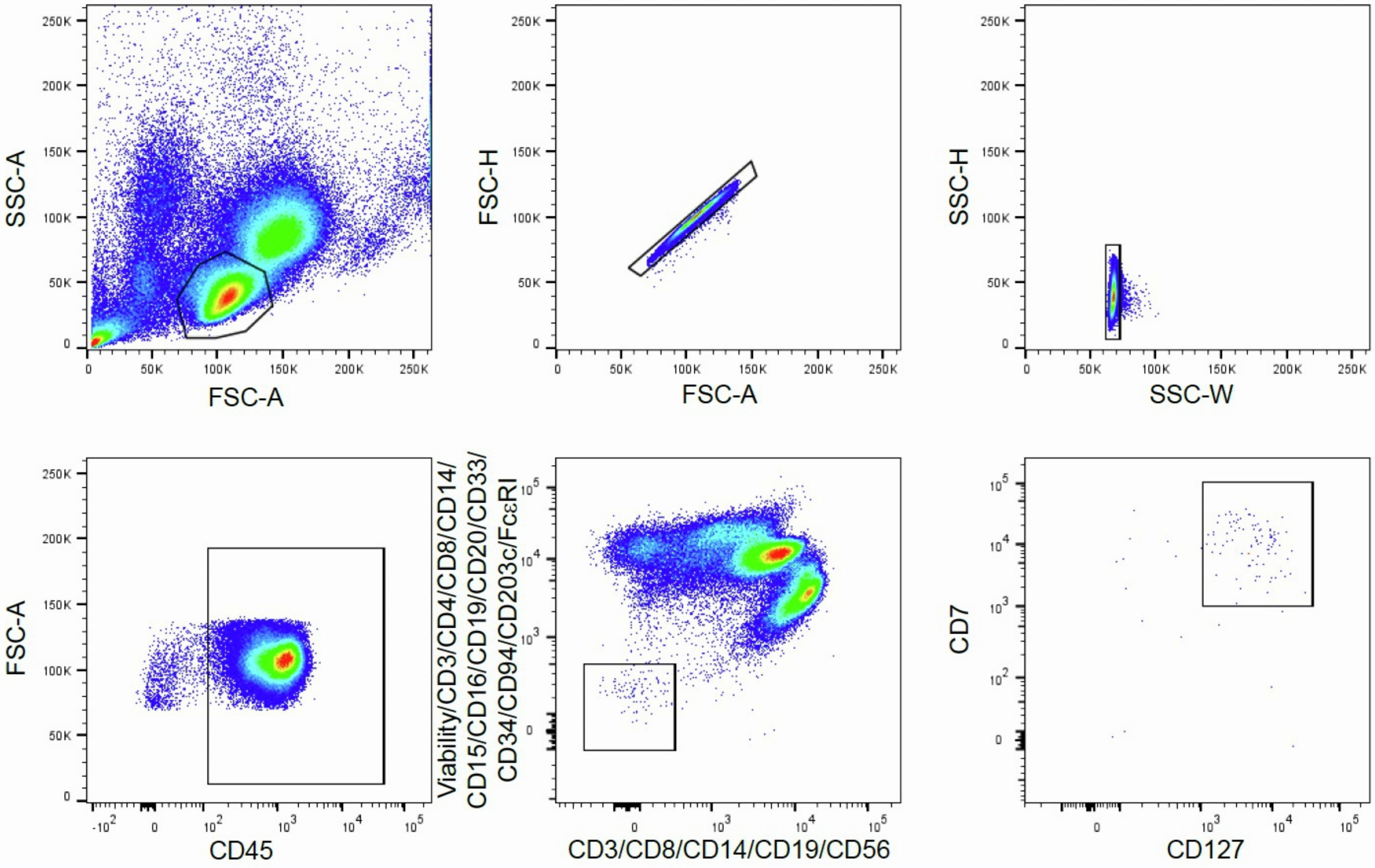

Figure S6. Representative gating strategy for the sorting of ILCs for sc analyses, Related to STAR Methods "Cell sorting and flow cytometry".
